# The Mcgill thyroid nodule score – does it help with indeterminate thyroid nodules?

**DOI:** 10.1186/s40463-015-0058-6

**Published:** 2015-02-03

**Authors:** Rickul Varshney, Veronique-Isabelle Forest, Marco A Mascarella, Faisal Zawawi, Louise Rochon, Michael P Hier, Alex Mlynarek, Michael Tamilia, Richard J Payne

**Affiliations:** McGill University, Otolaryngology-Head and Neck Surgery, Royal Victoria Hospital, 687 Pine Ave. West, E3-37, Montreal, QC H3A 1A1 Canada; King Abdulaziz University, Jeddah, Saudi Arabia; Department of Pathology, Jewish General Hospital, McGill University, 3755 Côte-Ste-Catherine Road, Montreal, QC H3T 1E2 Canada; Department of Endocrinology and Metabolism, Jewish General Hospital, McGill University, 3755 Côte-Ste-Catherine Road, Montreal, Canada

**Keywords:** Thyroid cancer, McGill Thyroid Nodule Score, Indeterminate nodule, Ultrasound guided fine-needle aspiration

## Abstract

**Background:**

Ultrasound guided fine-needle aspiration (USFNA) biopsy of thyroid nodules often gives a result of indeterminate pathology, placing thyroid specialists in difficult management situations. The aim of this study is to evaluate the incidence of malignancy in patients undergoing surgery and to correlate these results with the McGill Thyroid Nodule Score (MTNS).

**Methods:**

We performed a retrospective study comparing USFNA results, MTNS and histopathology of patients undergoing thyroid surgery between 2010 and 2012. Pre-operative USFNA results were divided into three subgroups: benign, indeterminate and suspicious for/malignant. The indeterminate USFNA subgroup comprised of Bethesda type III (atypia of undetermined significance) and Bethesda type IV (follicular neoplasms, including Hurthle cell neoplasms) lesions. Post-operative histopathology was divided into benign or malignant groups.

**Results:**

Of the 437 patient charts reviewed, 57.0% had an indeterminate USFNA biopsy. Within the indeterminate group, the malignancy rate was 39.8%. For indeterminate USFNA, the median MTNS was 7 (32% risk of malignancy) for benign nodules and 9 (63% risk of malignancy) for malignant nodules on post-operative histopathology (*p < 0.05*).

**Conclusion:**

The rate of malignancy in operated patients with an indeterminate USFNA result was 39.8%. The MTNS can be of value to thyroid specialists in pre-operative decision-making when dealing with an indeterminate result of a thyroid nodule on USFNA.

## Background

**Figure 1:**
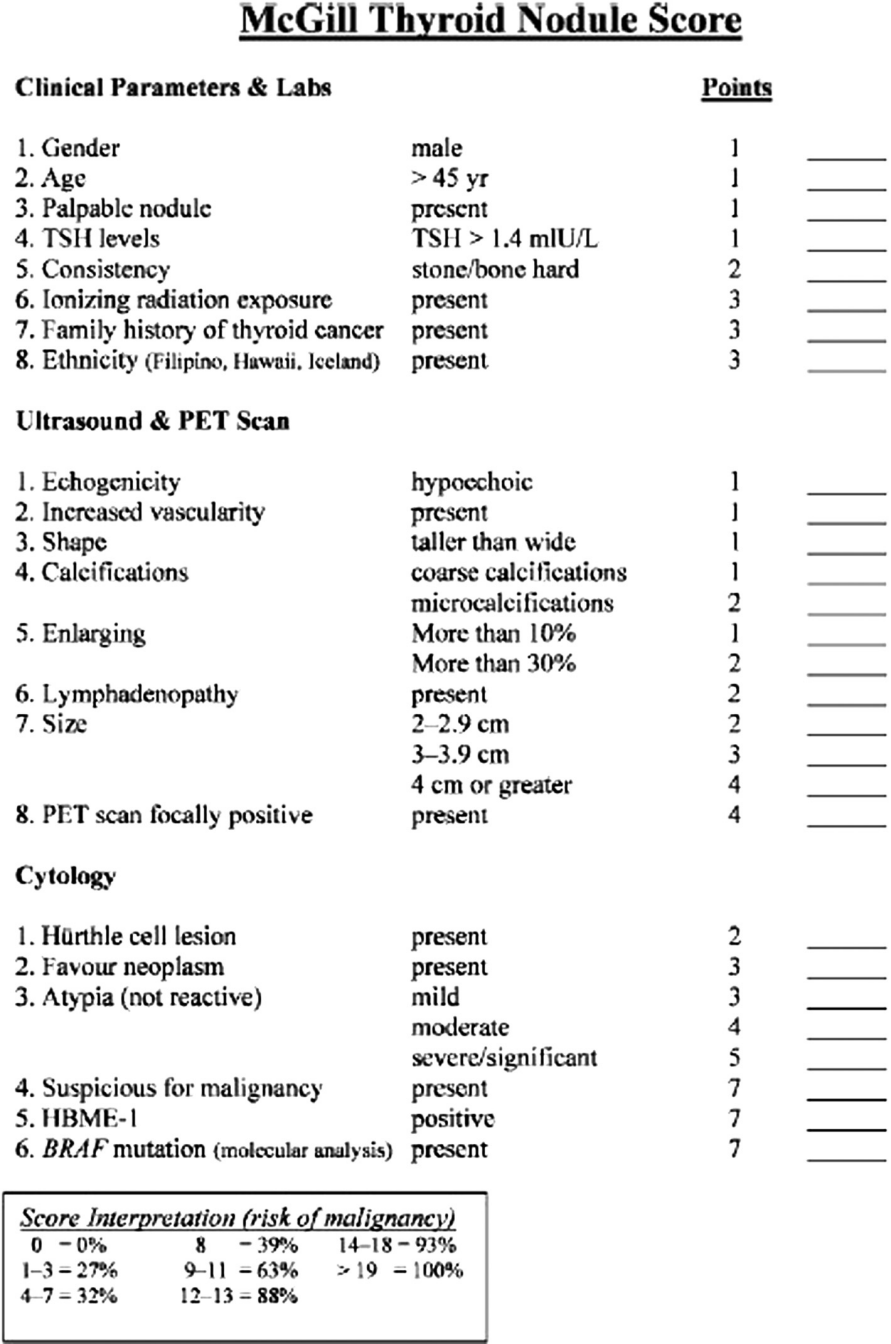
**The McGill Thyroid Nodule Score (MTNS).** PET: positron emission tomography; TSH: Thyroid Stimulating Hormone.

Ultrasound guided fine-needle aspiration (USFNA) biopsy is the first-line modality in the work-up of thyroid nodules [1,2]. Biopsy results are often categorised into three main groups: benign, malignant or indeterminate, with an indeterminate USFNA result posing diagnostic and management dilemmas. Given that 15% to 48% of thyroid nodule biopsies are indeterminate [2-7], this is not an infrequent situation for thyroid specialists to encounter. In order to estimate the risk of malignancy of a thyroid nodule, the McGill Thyroid Nodule Score (MTNS) was developed. This scoring system combines patient history, demographics, imaging and USFNA results [8]. Each of the 22 variables in the MTNS are assigned a weighted, relative point score based on the robustness of the current supporting evidence for that risk factor (Figure 1). The MTNS has been shown to correlate with the risk of malignancy in a large series of patients [8].

In this study, our objective was to evaluate the rate of indeterminate nodules and incidence of malignancy among these patients undergoing thyroid surgery at our center. Next, we aimed to assess whether the MTNS can help predict the likelihood of malignancy in such patients with indeterminate cytology.

## Methods

We performed a retrospective review of 559 consecutive patients who underwent thyroid surgery at the McGill University teaching hospitals between 2010 and 2012. The study received approval by the hospitals’ Research Ethics Committee. Of the 559 patients, 122 were excluded as a MTNS was not calculated pre-operatively or had an unsatisfactory USFNA result (Bethesda type I). As a result, a total of 437 patients were analyzed. Of note, patients with an indeterminate USFNA biopsy who did not undergo surgery were excluded from the study.

The patients analyzed were divided into three groups based on pre-operative USFNA diagnosis: benign (Bethesda type II), indeterminate (Bethesda types III-IV), and suspicious for malignancy/malignant (Bethesda types V-VI). The Bethesda type IV lesions included both follicular neoplasms/suspicious for follicular neoplasms and Hurthle cell neoplasms. Post-operative diagnoses were divided into benign and malignant based on histopathology. All cases of malignancy were included: follicular, papillary, medullary and anaplastic carcinoma. Demographic profiles, MTNS scores, USFNA results and post-operative histopathology were noted for each patient. An experienced thyroid cancer pathologist reviewed all histological specimens.

For data analysis, six subgroups of patients were created: (1) benign and indeterminate USFNA (Bethesda type II, III, IV) with benign final diagnoses; (2) benign and indeterminate USFNA (Bethesda type II, III, IV) with malignant final diagnoses; (3) benign USFNA (Bethesda type II) with benign final diagnoses; (4) benign USFNA (Bethesda type II) with malignant final diagnoses; (5) indeterminate USFNA (Bethesda type III and IV) with benign final diagnoses; (6) indeterminate USFNA (Bethesda type III and IV) with malignant final diagnoses. Median and mean MTNS scores were calculated for each group. We compared group 1 with 2, group 3 with 4 and group 5 with 6 in order to determine if the MTNS allows for a better prediction of malignancy when faced with an indeterminate USFNA result. SPSS 20.0 was used for statistical analysis using the independent *t*-test. A *p < 0.05* was considered to indicate statistical significance.

## Results

There were 437 patients analyzed in this study, 181 (39 male, 142 female) with a benign final diagnosis and 256 (54 male, 202 female) with a malignant final diagnosis. The average patient age was 53.3 (±16.1) years old in the benign group and 55.1 (±16.4) years old in the malignant group. There was no statistical difference in age or gender between groups (Table 1).

**Table 1:** **Patient demographics distributed into post-operative benign or malignant diagnoses for all patients**

		**Benign**	**Malignant**	***p-value***
**Gender**	Male	39 (21.5%)	54 (21.1%)	*p > 0.05*
	Female	142 (78.5%)	202 (78.9%)	
**Age**		53.2 (±16.1)	55.1 (±16.4)	*p > 0.05*

The pre-operative USFNA classification was as follows: 33 (7.6%) benign (Bethesda type II), 249 (57.0%) indeterminate (Bethesda type III and IV) and 155 (35.5%) malignant/suspicious for malignancy (Bethesda type V and VI). Post-operatively, the malignancy rate was 58.6% (see Table 2).

**Table 2:** **Distribution of FNAB diagnosis and post-operative pathology diagnoses**

**FNAB results**	**Patients with benign post-operative pathology n (%)**	**Patients with malignant post-operative pathology n (%)**
Benign (Bethesda II)	25 (75.8%)	8 (24.2%)
Indeterminate (Bethesda III, IV)	150 (60.2%)	99 (39.8%)
Malignant/Suspicious for malignancy (Bethesda V, VI)	6 (3.8%)	149 (96.1%)
Whole population (Bethesda, II, III, IV, V, VI)	181 (41.4%)	256 (58.6%)

The median and mean MTNS for each subgroup of patients is presented in Table 3. The corresponding estimated risk of cancer using the MTNS is also provided; a percentage is conferred to each score. The average MTNS was 7 for the benign final diagnosis group and 9 for the malignant final diagnosis group (*p <0.001*). These MTNS values conferred a pre-operative estimated risk of having a malignant nodule of 32% in the benign group and 63% in the malignant group. Similarly, the median MTNS for patients with an indeterminate USFNA result with benign final pathology was also 7 whereas it was 9 for patients with malignant disease post-operatively (*p = 0.001*).

**Table 3:** **Median and mean MTNS scores for each subgroup of patients**

**FNAB**	**Post-operative diagnosis**	**Median MTNS (Mean)**	**Estimated risk of cancer**	***p-value***
**Whole population (Bethesda II, III, IV)**	Benign	7.0 (7.76)	32%	*p <0.001*
	Malignant	9 (9.38)	63%	
**Benign (Bethesda II)**	Benign	6 (6.68)	32%	*p = 0.428*
	Malignant	5 (4.50)	32%	
**Indeterminate (Bethesda III, IV)**	Benign	7 (7.94)	32%	*p = 0.001*
	Malignant	9 (9.78)	63%	

p <0.05 considered significant.

## Discussion

USFNA is an integral part in the work-up of thyroid nodules [1,2]. When an USFNA biopsy indicates an indeterminate result, there is often uncertainty in the risk of malignancy. According to some authors, up to 74% of patients with indeterminate nodules undergo surgery [3] largely due to the limitations of USFNA. However, not all indeterminate nodules are malignant. Tutuncu et al. reported an incidence of 33.3% and 23.0% for malignancy in Hurthle cell lesions and follicular lesions, respectively [9]. Similarly, Sugino et al. demonstrated a 28% incidence of malignancy in their indeterminate pathology group [10]. Notably, these studies included only patients undergoing thyroid surgery. However, the risk of malignancy needs to be weighed against the 2 to 10% risk of serious surgical complications [5] such as recurrent laryngeal nerve injury, hypocalcemia, as well as the lifelong need for thyroid hormone replacement.

Our study shows that 57.0% of patients with pre-operative USFNA results are indeterminate (Bethesda III and IV), obscuring clinical management. This rate of indeterminate nodules is higher than that reported in the literature, but rates as high as 48% have been reported [7]. This is likely due to a selected population of patients undergoing surgery. At our institution, the routine use of HBME staining is not performed and may further explain the high rate of indeterminate nodules. Moreover, the categories of atypia of undetermined significance (AUS, Bethesda III) and follicular lesion of undetermined significance/Hurthle cell lesion of undetermined significance (FLUS/HUS, Bethesda IV) are often reported with a wide range of rates in the literature [11-13] due to the heterogeneity of their characteristics and subjectivity in the diagnostic criteria. Thus, it was deemed appropriate to analyze these biopsy results together when assessing malignancy risk and the MTNS until more robust histologic determinants and validated scoring systems are established.

For patients with an indeterminate USFNA result (Bethesda type III and IV), the median MTNS for all patients with benign disease post-operatively was 7 (32% risk of malignancy) and 9 for patients with malignant disease (63% risk of malignancy). Although many would argue that even a 32% risk of malignancy is considered significant and requires action, the difference lies in the mode of intervention to be chosen. In cases of a MTNS of 7, a diagnostic hemi-thyroidectomy would most likely be the therapeutic option chosen. With a MTNS of 9, a higher risk of thyroid cancer likely exists. In such cases, a discussion with patients about the utility of a total thyroidectomy may be advocated, precluding the need for completion thyroidectomies in cases of malignancy. Despite this, it remains unclear of the significance of an indeterminate USFNA with a MTNS score of 8.

With regards to malignancy rates in our population, the malignancy rate for benign (Bethesda II), indeterminate (Bethesda III-IV) and suspicious for malignancy/malignant (Bethesda V-VI) were 24.2%, 39.8% and 96.1%, respectively. This is in line with results by Al-Shraim et al. who reported rates of 10.3%, 25.0% and 86.4%, respectively [11]. Our 39.8% malignancy rate in the indeterminate group is also comparable to reports by Granados-Garcia et al. who showed a 40% rate of malignancy [14], Rossi et al. who reported a 35.7% risk of malignancy [15] and Lee et al. who demonstrated a 46.3% incidence of cancer for indeterminate nodules [2]. The high rate of indeterminate nodules along with a significant incidence of malignancy within this population reinforces the need for ancillary tools to help guide management.

For these reasons, there has been a rise in diagnostic tests available to thyroid specialists in the past few years. One such tool is the 4-gene classifier (BRAF, RET/PTC, PAX8/PPAR_ϒ_, and RAS) [16]. Genetic markers have a high specificity and positive predictive value [5] and can improve diagnostic accuracy. However, they have a low sensitivity and negative predictive value [5,16]. A more recent advancement is the Afirma gene expression classifier (Veracyte, Inc., San Francisco, USA) which measures mRNA transcript expression levels of 142 genes. It has shown to have a high sensitivity with moderate specificity [5]. Nonetheless, a major obstacle to their widespread use is the high costs [6] associated with such tests.

Another tool available to guide clinical management of thyroid nodules is MTNS [8]. This non-validated scoring system uses evidence-based risk factors for thyroid cancer to estimate the risk of malignancy. It is unique as it does not solely rely on cytology results. The MTNS is based on risk factors for thyroid cancer categorized into 8 clinical/laboratory parameters, 8 imaging features (ultrasound/positron emission tomography scan) and 6 histopathological criteria (Figure 1). These risk factors are based on the American Thyroid Association guidelines as well as a literature review on thyroid malignancy risk factors. It was developed by a multidisciplinary committee at the McGill University. Sands et al. demonstrated a PPV and specificity for carcinoma of 66% and 13% for scores >4, 81% and 66% for scores >8, 96% and 96% for scores >14 and 100% and 100% for scores >19, respectively [8].

Certain factors limit the generalization of this study. Firstly, this is a retrospective review of patients undergoing thyroid surgery with an indeterminate USFNA. Inherently, these patients may be at an increased risk of malignancy. Secondly, not all patients seen at our institution have a calculated MTNS, resulting in patients being excluded from analysis. Also, our difference of 2 points on the MTNS, though statistically significant, must be retain as an average difference when applied in clinical practice. As such, a small difference in MTNS can predict for a benign or malignant nodule, changing therapeutic options for individual patients.

Modern diagnostic tests to help direct the need for and extent of surgery, particularly in patients with indeterminate cytology are needed. Furthermore, validated risk stratification algorithms, similar to the MTNS, are useful in the interim until such tests are widely available.

## Conclusion

Our study shows that when looking at indeterminate thyroid nodules, the MTNS is able to demonstrate an increased pre-operative risk of carcinoma for nodules found to be malignant. Based on these results, we suggest that the MTNS be considered as a tool to guide the management of indeterminate thyroid nodules, as it not only refines the pre-operative estimated risk of cancer, but also clarifies communication between physicians and patients.

## Abbreviations

FNA: Fine needle aspiration
MTNS: McGill Thyroid Nodule Score
USFNA: Ultrasound guided fine needle aspiration
